# Lauric acid with or without levodopa ameliorates Parkinsonism in genetically modified model of *Drosophila melanogaster* via the oxidative–inflammatory–apoptotic pathway

**DOI:** 10.1002/brb3.70001

**Published:** 2024-09-08

**Authors:** Olumayowa K. Idowu, Olufunke O. Dosumu, Ayodeji S. Boboye, Ademola A. Oremosu, Abdullahi A. Mohammed

**Affiliations:** ^1^ Department of Anatomy, College of Medicine University of Lagos Lagos Nigeria; ^2^ Department of Anatomy, College of Health Sciences Osun State University Osogbo Nigeria; ^3^ Department of Anatomy, College of Health Sciences Federal University of Technology Akure Nigeria; ^4^ Department of Human Anatomy, School of Medicine and Pharmacy College of Medicine and Health Sciences University of Rwanda Butare Rwanda

**Keywords:** apoptosis, dopa decarboxylase, *Drosophila melanogaster*, lauric acid, neuroinflammation, Parkinson's disease, α‐synuclein

## Abstract

**Background:**

Parkinson's disease (PD), the most prevalent type of Parkinsonism, is a progressive neurological condition characterized by a range of motor and non‐motor symptoms. The complicated etiology of PD is thought to involve a summation of aging, genetic predisposition, and environmental variables. However, the α‐synuclein protein plays a significant role in the disease's pathophysiology.

**Materials and methods:**

The UAS‐α‐Syn and Ddc‐Gal4 strains were crossed to produce offspring referred to as PD flies. The entire population of flies was divided into five groups, each having about 100 flies and five replicates. The control group (w^1118^) and the PD group not receiving treatment were exposed to lauric acid (LA)/levodopa (LD)‐free diet, while the PD groups that received treatments were fed with either a 250 mg/kg LA diet, a 250 mg/kg LD diet, or a combination of the two for 21 days. Longevity, geotaxis, and olfactory assays were performed in addition to other biochemical tests.

**Results:**

As a result of the overexpression of α‐synuclein, the locomotive capacity, lifespan, and antioxidant status were all significantly (*p* < .05) reduced, and the apoptotic and neuroinflammatory activities were increased. Nevertheless, the majority of the treated flies improved significantly (*p* < .05).

**Conclusion:**

LA, whether combined with LD or not, elicited a significant response in α‐synuclein/dopa decarboxylase genetically modified *Drosophila melanogaster* Parkinsonism models.

## Graphical abstract

Lauric acid exhibits therapeutic potential in alpha‐synuclein‐mediated Parkinsonism by mitigating behavioral impairment, reducing oxidative stress, and curbing neuroinflammation. It also appears to counteract apoptosis and enhance neuronal longevity. These effects collectively suggest lauric acid could be a promising adjunctive treatment for Parkinson's disease.

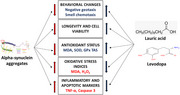

## 1 INTRODUCTION

Parkinson's disease (PD) is a spectral clinical condition that includes motor dysfunction such as tremors, bradykinesia, stiffness, gait issues, and balance issues, as well as several non‐motor symptoms such as loss of smell, constipation, depression, and rapid eye movement sleep behavior disorder (Adam et al., 2023; Ongari et al., 2024). Neurodegeneration, vascular injury, structural lesions, infections, and toxicant exposure (Cersosimo & Koller, 2006; Zagaria, 2007), progressively escalating the loss of striatal dopamine in the substantia nigra and pars compacta, are considered causal for the onset of PD (Armstrong & Okun, 2020; Cersosimo & Koller, 2006). More so, ageing, genetic predisposition, and environmental factors are regarded as contributors to the aggregation of α‐synuclein in dopamine neurons of PD (Haque et al., 2022; Pang et al., 2019). Despite scientific contributions to the understanding of the mechanism underlying the onset and progressing severity of PD, the precise process by which α‐synuclein destroys dopamine neurons still eludes us. Evidence from clinical and preclinical studies has revealed that the accumulation of reactive oxygen species (ROS) and mitochondria dysfunction are major hallmarks for the aggregation of α‐synuclein, cellular dysfunction, and cell death of dopaminergic neurons in PD (Borsche et al., 2021; Haque et al., 2022; Kung et al., 2021).

The expression of features of PD including shortened lifespan, loss of dopaminergic neurons, motor function deficits, and oxidative stress has been reported in the offspring from the crosses between α‐synuclein‐UAS and Ddc‐Gal4 strains (Ishola et al., 2023; Liu et al., 2022). Overexpression of the SNCA transgene in *Drosophila melanogaster* (*Drosophila*) downregulates antioxidant enzymes activities, thereby increasing the level of ROS and overexpression of human α‐synuclein to the neurons of the flies (Jahromi et al., 2021). In a two‐cohort study of 245 participants, inflammation due to impaired mitophagy and accumulation of inflammatory cytokines was linked to the onset and progressiveness of idiopathic and genetic predisposition (PRKN/PINK1) of PD (Borsche et al., 2020).

Further, the release of inflammatory cytokines, alteration in molecular pathways, and factors that may impede both intrinsic and extrinsic apoptotic processes of the dopaminergic neurons in the substantial nigra and cortex of the brain have been linked to the detrimental effects of PD. In dopaminergic neurons, impairment in the level of tumor necrosis factor‐α (TNF‐α) has been shown to trigger downstream apoptosis mediated by caspase‐3 activation (Abdelkader et al., 2020) and accumulation of malondialdehyde (MDA), a product of lipid peroxidation (Naghipour et al., 2020). Brain microglia and astrocytes being the major resident macrophages of the central nervous system (CNS) are easily triggered in response to neuroinflammation and damages due to oxidative stress (O. K. Idowu, Oluyomi, et al., 2022; Isik et al., 2023; Ward et al., 2022). In PD, hyperactivation of microglia cells results in harmful release and accumulation of ROS, hydrogen peroxide (H_2_O_2_), and TNF‐α, thereby possibly contributing to neuronal death and severities associated with PD and making the management more challenging (Dhankhar et al., 2020; Farooqui & farooqui, 2011).

The gold standard medication for treating PD motor symptoms is levodopa (LD). It is recognized to have the best improvement in motor function when compared to other treatments (Urso et al., 2020). Dopamine concentrations in the brain are raised as a result of LD's ability to penetrate the blood‐brain barrier and be converted to dopamine (Qu et al., 2018). Despite the therapeutic efficacy of LD, several side effects including dyskinesia and erratic involuntary movements have been associated with its long usage; hence, scientific attention for optimal therapy for PD is increasing (Ghadery et al., 2022).

Ketogenic diet is becoming more popular as therapy for several neurological and neurodegenerative conditions (Pietrzak et al., 2022). Emerging studies have suggested that ketone bodies might exert a neuroprotective influence by acting as a substantial energy source for neurons, enhancing mitochondrial biogenesis, and diminishing oxidative stress (Yang et al., 2019). Lauric acid (LA) is a medium‐chain fatty acid, which can be found in a variety of vegetable oils and fats, as well as in human breast milk (Dubo et al., 2019; Kour et al., 2020). LA is metabolized in the hepatic mitochondria releasing acetyl‐coenzyme‐A for the formation of ketone bodies (McCarty & DiNicolantonio, 2016). Notably, there is a growing body of evidence connecting the neuroprotective functions of LA to the generation of ketone bodies (Mett & Müller, 2021). In their study, Zhou et al. (2023) highlighted the therapeutic effects of LA against cellular damage and apoptosis induced by peroxides. They attributed these effects to LA's ability to inhibit ROS production and improve mitochondrial function. In a mouse model of Alzheimer's disease, LA improved cognitive impairment by clearing the accumulation of amyloid proteins in the hippocampus and suppressing harmful hyperactivation of astrocytes and microglia (Kumar et al., 2022). Additionally, LA inhibits the mRNA overexpression of apolipoprotein, lowers levels of low‐density lipoprotein, raises levels of high‐density lipoprotein, and thus lowers total cholesterol levels (Arunima & Rajamohan, 2018).

Furthermore, it has been previously documented in the literature that ketone bodies work as neuroprotective agents and guard against damage to tyrosine hydroxylase positive and substantia nigra's dopaminergic neurons (Cheng et al., 2009; Kashiwaya et al., 2000). According to Veech et al. (2001), dopamine therapy with the addition of ketone bodies may be able to prolong the period during which dopamine is effective. This study is aimed at investigating the effect of LA with LD co‐treatment on α‐synuclein transgenic *Drosophila* model of PD.

*Drosophila* is recognized to replace the usage of higher animals in biomedical research because it has distinctive biological characteristics comparable to those of vertebrates (Lehmann, 2018; Na et al., 2013). According to genomic studies, *Drosophila* has orthologs for over 75% of human disease genes (Hirth, 2010). The usage of *Drosophila* as a PD model is steadily growing due to its lower ethical concerns and validation by the European Center for the Validation of Alternative Models and ability to produce robust data (Feany & Bender, 2000; Hirth, 2010).

## 2 MATERIALS AND METHODS

### 2.1 Chemicals

All compounds and reagents used for the study were of analytical grade. LD (99% purity) and LA (99% purity) were purchased from Shaanxi Phoenix Tree, Biotech Co., Ltd. and Xi'an Sonwu Biotech Ltd., China, respectively. All other reagents and chemicals used were purchased locally.

### 2.2 *Drosophila* stock and culture

Genetically manipulated strains of *Drosophila* w^1118^ (#3605), w[*]; P{w[+mC] = UAS‐Hsap∖SNCA.F}5B (#8146) and w[1118]; P{w[+mC] = Ddc‐GAL4.L}Lmpt[4.36] (#7009) of Bloomington *Drosophila* stock center origin, were gifted by Dr. Ismaila O. Ishola of the Department of Pharmacology and Therapeutics, University of Lagos, Nigeria. The fly strains were bred in the Drosophila Laboratory, Department of Anatomy, University of Lagos, Nigeria, on a cornmeal diet mixed with 1% w/v agar, 1% w/v brewer yeast, and 0.08% v/w nipagin, at a constant temperature range of 22–24°C and 60%–70% range of relative humidity, under 12 h darkness and light cycle.

### 2.3 Strain crosses

The fly interstrain crossing was conducted using the procedure outlined in a previous study by O. Idowu, Dosumu et al. (2022). Within 1–3 h of emerging from the vials, females of the Ddc‐Gal4 strains were checked for virginity and removed to new vials. UAS‐α‐Syn strain males were then exposed to the virgin females of the Ddc‐Gal4 at a ratio of one to three (1:3). The flies that resulted from these crossings express an aggregation of human α‐synuclein in dopaminergic and serotonergic neurons, and are thus called the PD flies (D'Souza et al., 2022; O. Idowu, Dosumu, et al., 2022).

### 2.4 Research design

The approach described by Dosumu et al. (2021) with minor modifications was used to calculate the optimal LA dose. 1‐ to 3‐day‐old PD flies were divided into four groups of 20 flies each, and they were given 14 days of exposure to LA at doses of 0, 125, 250, and 500 mg/kg diet. Daily mortality was tracked, and the survivors underwent a geotaxis test.

Following the dose determination, fly samples that were between 1 and 3 days old were randomly sorted into five groups (control, PD, PD + LA, PD + LD, and PD + LA + LD), each containing about 100 flies and five replicates. The LA/LD‐treated PD groups, PD + LA, PD + LD, and PD + LA + LD, were exposed to 250 mg LA per kg diets (determined from pilot study), 250 mg LD per kg diets (Ayikobua et al., 2020), and a combination of the two treatment regimens, respectively. The control group and the PD group not receiving treatment were exposed to LA/LD‐free diet. Throughout 21 days, the flies were fed a freshly produced diet every other day. After undergoing negative geotaxis and olfactory tests, the flies were given a fly nap to put them to sleep before the heads were harvested, weighted, homogenized in 0.1 M phosphate‐buffered saline at pH 7.2 in a ratio of 1 mg:10 µL, and freeze‐centrifuged (4°C) at 2500 rmp for 15 min (Abolaji et al., 2018). The supernatant from the homogenate was then collected into labeled Eppendorf tubes and was used to measure various neurochemical parameters. A survival assay was also conducted which lasted until the last fly was dead.

### 2.5 Survival assay

The survival assay method reported by Farombi et al. (2018) was used to measure longevity. Daily death event was assigned a score of one (1), while a score of zero (0) was assigned when there was no death event. Records were taken throughout the experiment until the last fly was dead, and the data were plotted on survival curve.

### 2.6 Negative geotaxis test for locomotor function

Twenty flies were placed in vertical tubes from each group and allowed to acclimate for 15 min. Following that, the test tube was turned upside down, and the flies were gently tapped to the bottom. The number of flies that ascended the tube in less than 8 s to a height of 17.5 cm was counted. The performance index was determined by averaging the highest fly count relative to all fly counts. This technique was performed three times at 1‐min interval, and the data were recorded for analysis (Vang et al., 2012).

### 2.7 Smell chemotaxis test for olfactory function

Twenty flies were placed in an empty 20‐cm‐long test vial for 15 min to acclimatize prior to the test. A repellant (100 mM benzaldehyde) dissolved in 1.5% agar was attached to the vial at one end. After that, the flies were gently tapped in the direction of the fly repellant and slanted. The number of flies in the farthest third of the test tube away from the repellant was counted within 1 min. The average percentage of all flies in the area farthest from the repellent was used to compute the repulsive index. Data were recorded for analysis after the test was conducted three times with a 1‐min interval between each repetition (Vang et al., 2012).

### 2.8 Determination of brain MDA level

Using a modified version of the method described by Karatas et al. (2002), the supernatant from the homogenate was analyzed by spectrophotometry to determine the amounts of MDA. The flies' eyes were removed to stop the pigment in the eyes from influencing MDA absorbance. Results were calculated as µmol/mL.

### 2.9 Determination of brain hydrogen peroxide level

The H_2_O_2_ level was evaluated using the procedure outlined by Wolff (1994). The samples were added to the reaction mixture along with FOX 1 (100 mM xylenol orange, 50 mL of 250 mM ammonium ferrous sulfate, 10 mL of 100 mM sorbitol, 5 mL of 25 mM H_2_SO_4_, and 30 mL of distilled water). After 30 min of room temperature incubation, the absorbance at 560 nm was measured. By extrapolating the data from the standard curve, the numbers were changed from millimoles to µmol/mg protein.

### 2.10 Determination of brain superoxide dismutase activity

The capacity of superoxide dismutase (SOD) to suppress the epinephrine's self‐oxidation was assessed using the Sun and Zigma method (1978), which is based on the rise in absorbance at 480 nm as defined by Sun and Zigma. The reference cuvette was filled with 2.95 mL of buffer, 0.03 mL of the epinephrine substrate, and 0.02 mL of water. By tracking the shift in absorbance at 480 nm for 5 min, enzyme activity was estimated: ∑ = 4020 M^−1^ cm^−1^.

### 2.11 Determination of brain glutathione peroxidase activity

Using a Randox Ransel diagnostic kit (Randox Laboratories Ltd.), the activity of glutathione peroxidase (GPx) was determined as described by Paglia and Valentine (1967). Together with glutathione reductase and Nicotinamide Adenine Dinucleotide Phosphate Hydrogen (NADPH), GPx catalyzed the biochemical oxidation of glutathione by cumene hydroperoxide. Once the oxidized glutathione was reduced, NADPH was also oxidized to NADP. At 340 nm, the decrease in absorbance was recorded, and the amount of GPx activity was expressed as U/g protein.

### 2.12 Determination of total brain antioxidant status

Using the method outlined in the study by Biala et al. (2018), the total antioxidant status (TAS) in the brain homogenates was assessed using a Randox Ransel diagnostic kit (Randox Laboratories Ltd.). When metmyoglobin and hydrogen peroxide were added, the principle encouraged 2,2‐azino‐di‐(3‐ethylbenzthiazoline sulphonate) to create ABTS(*)(+). The hue of the ABTS(*)(+) is a comparatively constant blue‐green. At 600 nm, the antioxidant concentration in the homogenates was used to detect changes in the absorption, and mmol/L tissue is used to express the results.

### 2.13 Determination of cell mitochondrial metabolic rate (cell viability)

The mitochondrial metabolic rate of the flies was assessed using the enzymatic conversion of MTT reagent (3‐(4,5‐dimethylthiazol‐2‐yl)−2,5‐diphenyl‐2H‐tetrazolium bromide) to MTT formazan at a final concentration of 5 mg/mL. For 60 min at 37°C, the samples were incubated in MTT (500 µg/mL). The samples were then incubated in dimethyl sulfoxide (DMSO) for 30 min at 37°C after washing off excess MTT. The colored formazan salt was finally measured by spectrophotometry at 590 nm (Sudati et al., 2013; Ternes et al., 2014; O. K. Idowu et al., 2024).

### 2.14 Evaluation of the levels of caspase‐3 and tumor necrosis factor‐alpha

Using an ELISA kit (Elabscience), caspase‐3 activity and the expression of TNF‐α were assessed following the manufacturer's instructions. A Spectramax Plus 384 Microplate reader was used to calculate the absorbance at 450 nm.

### 2.15 Statistical analysis

**FIGURE 1:**
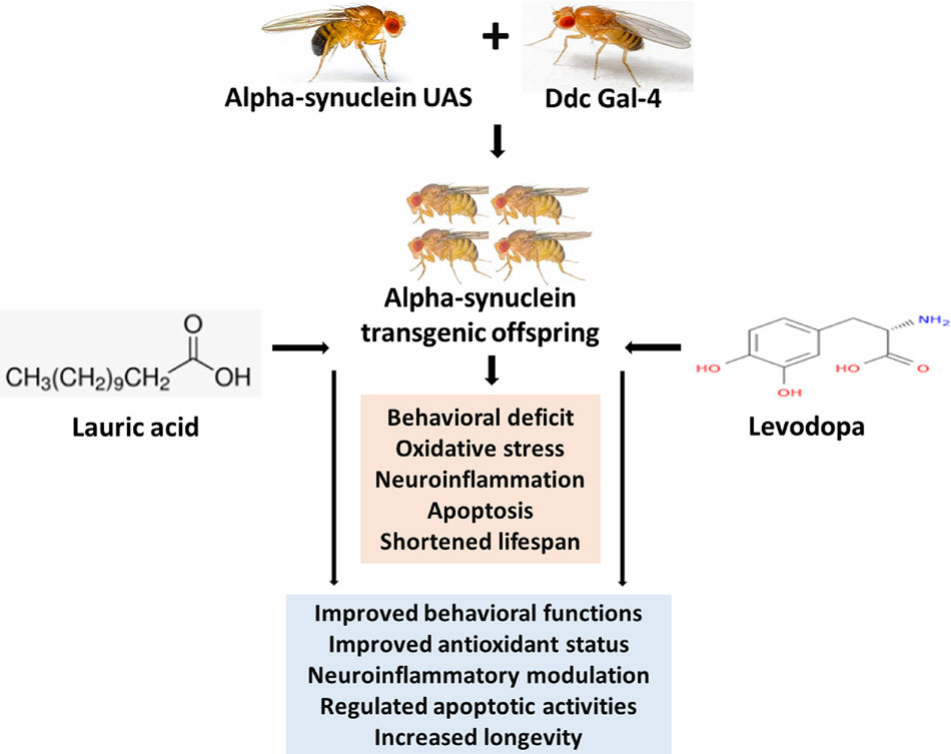
Schematic illustration of the research design, therapeutic effect of lauric acid, and levodopa on alpha‐synuclein transgenic *Drosophila melanogaster*.

All findings were shown as mean ± SEM. The statistical analysis and graphs were made using Graph Pad Prism 8 (Graph pad software Inc.). For multiple comparisons, the Tukey's test was employed in conjunction with a one‐way analysis of variance. *p* < .05 was used to determine significance Figure 1.

## 3 RESULTS

### 3.1 The effective dose of LA on Parkinsonism in *Drosophila* was determined

**FIGURE 2:**
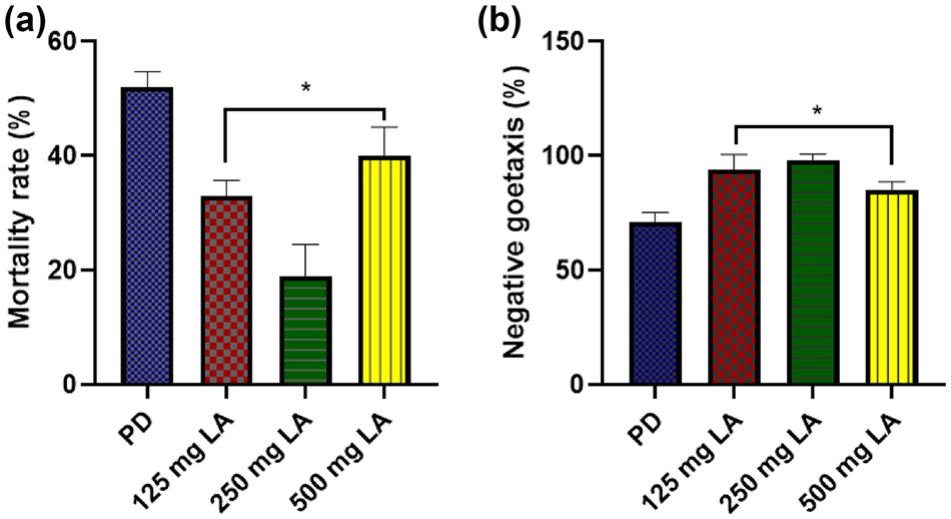
(a) Mortality rate on different doses of lauric acid and (b) geotaxis performance index on divided doses of lauric acid (LA). Data are presented as mean ± SEM. *p < .05 compared to the Parkinson's disease (PD) group.

Fly exposure to diets containing 125, 250, and 500 mg/kg of LA for 14 days resulted in a statistically significant (*p* < .05) decrease in mortality rates (*F* (3, 16) = 54.29, *p* = .0001) in all the treated groups and a significant (*p* < .05) improvement in geotaxis performance only in the group that received 250 mg/kg LA diet (*F* (3, 16) = 35.83, *p* < .0001) as compared to the PD group exposed to LA‐free diet (Figure 2a,b). For the main experiment, the diet containing 250 mg/kg LA was chosen because its administration resulted in the lowest mortality rate and the best geotaxis performance.

### 3.2 LA and LD improved locomotor and olfactory functions, and rate of survival in *Drosophila* model of Parkinsonism

**FIGURE 3:**
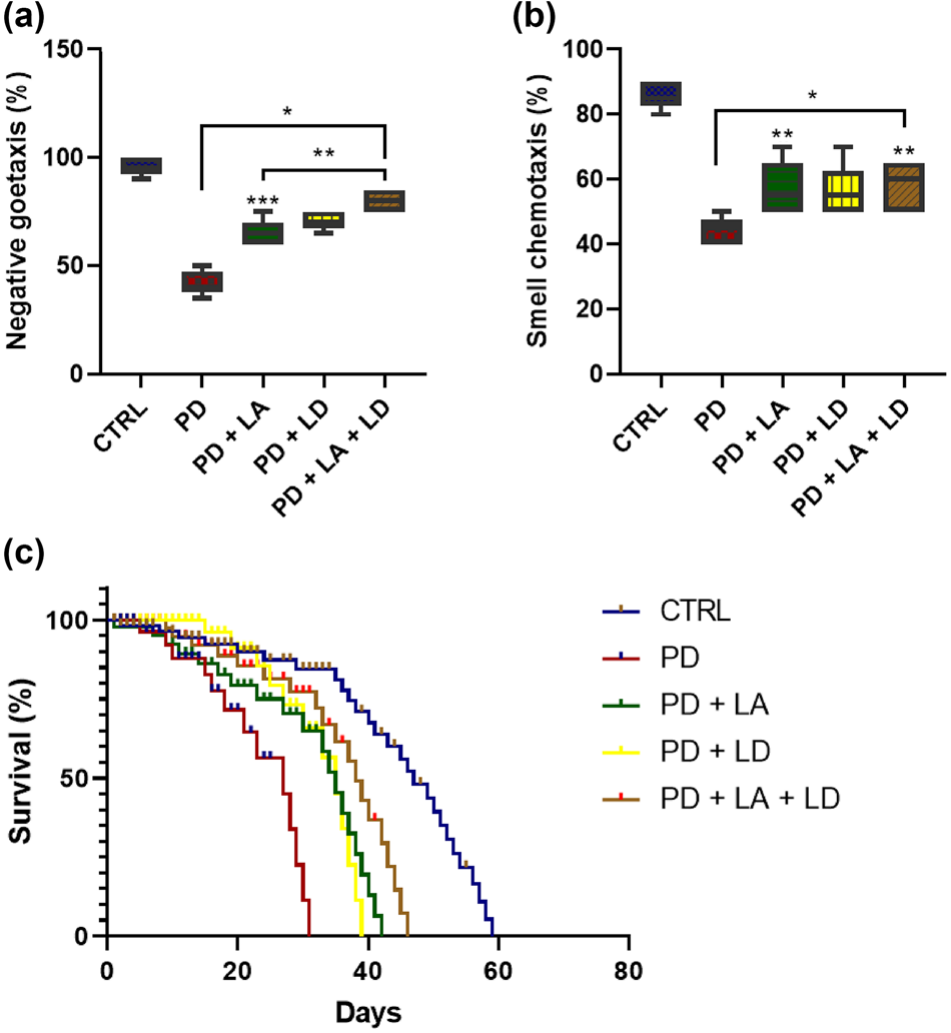
(a) Negative geotaxis, (b) smell chemotaxis, and (c) survival rate. Data are presented as mean ± SEM at *p < .05 compared to the control group; **p < .05 compared to the untreated PD group; ***p < .05 compared to PD + LD + LD. LA, lauric acid; LD, levodopa; PD, Parkinson's disease.

In comparison to the control, the results revealed a statistically significant (*p* < .05) decrease in geotaxis performance in all of the PD flies. However, in comparison to the PD group that was not treated, all PD groups that received LA and LD treatment demonstrated statistically significant (*p* < .05) improvement. Furthermore, the geotaxis performance of the PD + LA group was significantly (*p* < .05) lower than that of the PD + LA + LD group, although the PD + LD group showed no statistically significant difference compared to the PD + LA and PD + LA + LD groups (*F* (4, 20) = 76.10, *p *< .0001) (Figure 3a).

The outcomes of this study also showed that all of the PD flies had significantly lower olfactory ability when compared to the control (*p* < .05). However, the PD + LA and PD + LA + LD groups showed statistically significant (*p* < .05) improvement in contrast to the untreated PD group, but the PD + LD group did not. Moreover, there was no statistically significant difference across all the treated groups (*F* (4, 20) = 26.11, *p* < .0001) (Figure 3b).

The mean survival for the control, PD, PD + LA, PD + LD, and PD + LA + LD groups were 47, 27, 35, 35, and 38, respectively. When compared to the control, the mean survival for the PD, PD + LA, PD + LD, and PD + LA + LD groups were reduced by 11%, 6.6%, 6.6%, and 4.5%, respectively. Conversely, when compared to the PD group fed on LA/LD‐free diet, the mean survival for the PD + LA, PD + LD, and PD + LA + LD groups were increased by 4.4%, 4.4%, and 6.1%, respectively. Moreover, the PD + LA + LD group showed 1.7% higher survival than the PD + LA and PD + LD groups (Figure 3c).

### 3.3 LA and LD improved redox status and cell viability in the *Drosophila* model of Parkinsonism

**FIGURE 4:**
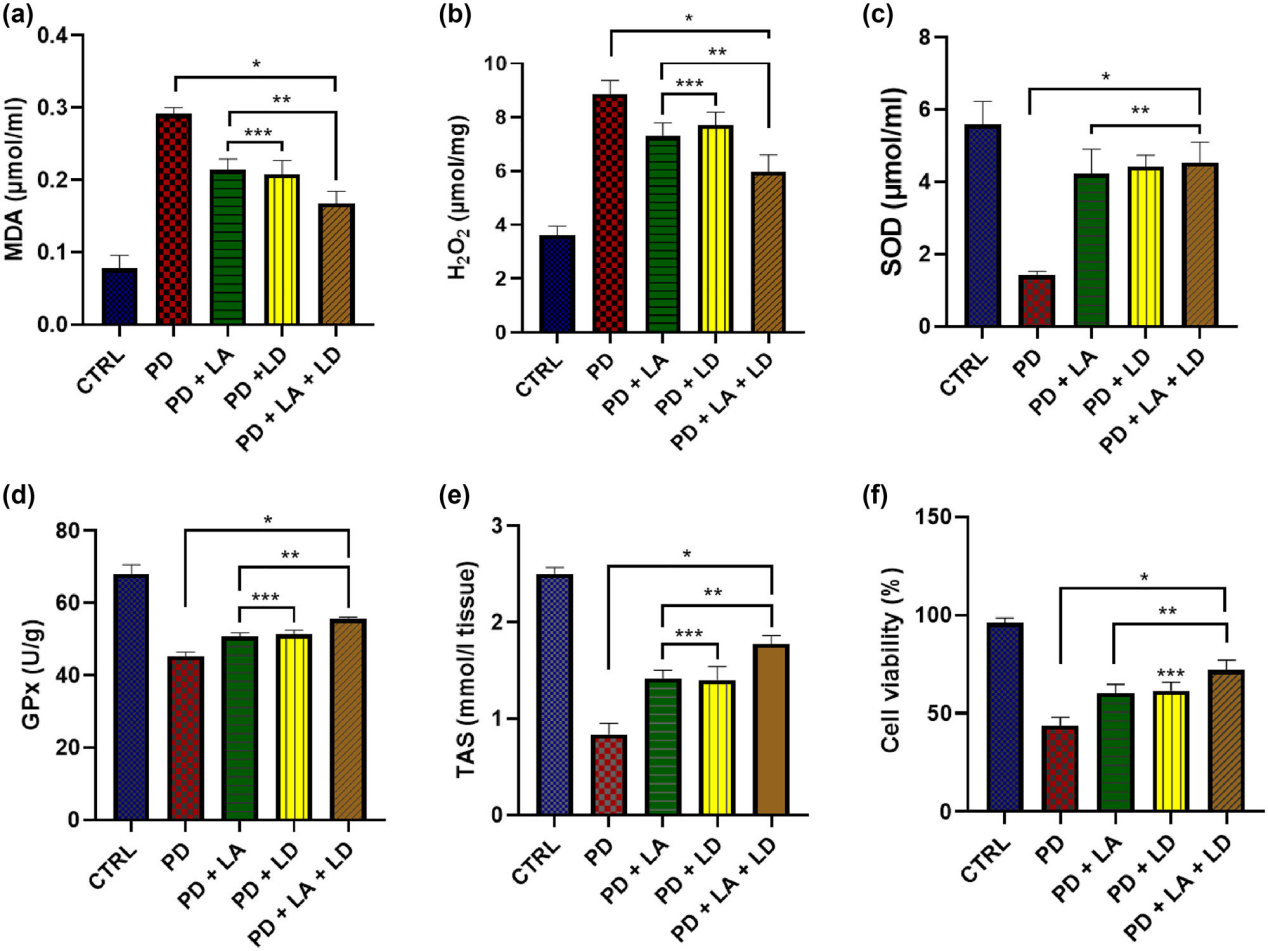
(a) Malondialdehyde (MDA) level, (b) hydrogen peroxide (H_2_O_2_) level, (c) superoxide dismutase (SOD), (d) glutathione peroxidase (GPx), (e) total antioxidant status (TAS), and (F) cell viability. Data are presented as mean ± SEM. *p < .05 compared to the control; **p < .05 compared to the untreated PD group; ***p < .05 compared to PD + LA + LD. LA, lauric acid; LD, levodopa; PD, Parkinson's disease.

All of the PD groups showed a statistically significant (*p* < .05) increase in MDA levels when compared to the control. On the other hand, compared to the PD group that was not treated with LA/LD, flies in all of the treated groups showed a significantly (*p* < .05) reduced MDA level. There was no statistically significant difference between the PD + LA and PD + LD groups, but the PD + LA and PD + LD groups both showed significantly (*p* < .05) lower levels of improvement than the PD + LA + LD group (*F* (4, 16) = 125.3, *p* < .0001) (Figure 4a).

When compared to the control, all of the PD groups displayed a statistically significant (*p* < .05) rise in H_2_O_2_ levels. In contrast, flies in all of the treated groups displayed a substantial (*p* < .05) decline in H_2_O_2_ level when compared to the PD group that was not treated with LA/LD. The PD + LA and PD + LD groups did not differ significantly from one another, whereas the PD + LA and PD + LD groups both displayed significantly (*p* < .05) reduced levels of improvement than the PD + LA + LD group (*F* (4, 16) = 67.41, *p* < .0001] (Figure 4b).

All the PD groups showed a statistically significant (*p* < .05) decrease in SOD levels in comparison to the control. However, compared to the untreated PD group, flies in all of the treated groups showed a significant (*p* < .05) increase in SOD level. Moreover, there was no statistically significant difference across all the treated groups (*F* (4, 16) = 42.58, *p* < .0001) (Figure 4c).

Comparing the GPx activity of all the PD groups to the control revealed a statistically significant (*p* < .05) decline. Flies in all of the treatment groups, however, displayed a significant (*p* < .05) rise in GPx activity when compared to the PD group that was not treated. Although the difference between them was not statistically significant, the PD + LA and PD + LD groups had a significantly (*p* < .05) lower activation of GPx than the PD + LA + LD group (*F* (4, 20) = 167.6, *p* < .0001) (Figure 4d).

There was a statistically significant (*p* < .05) decrease in TAS across all PD groups when compared to the control group. In contrast to the PD group that was not treated with LA/LD, however, flies in all of the treatment groups showed a significant (*p* < .05) increase in TAS. The PD + LA and PD + LD groups showed significantly (*p* < .05) lower TAS than the PD + LA + LD group, while the difference between the PD + LA and PD + LD groups was not statistically significant (*F* (4, 20) = 179.3, *p* < .0001) (Figure 4e).

In comparison to the control group, there was a statistically significant (*p* < .05) decrease in cell viability in all PD groups. On the other hand, flies in all treatment groups displayed a significant (*p* < .05) increase in cell viability as compared to the untreated PD group. While the PD + LA group did not substantially differ from the PD + LD and PD + LA + LD groups, the PD + LD group demonstrated considerably (*p* < .05) poorer cell viability than the PD + LA + LD group (*F* (1.887, 7.546) = 124.8, *p* < .0001) (Figure 4f).

### 3.4 LA and LD reduced the expression of markers for apoptosis and inflammation in the *Drosophila* model of Parkinsonism

**FIGURE 5:**
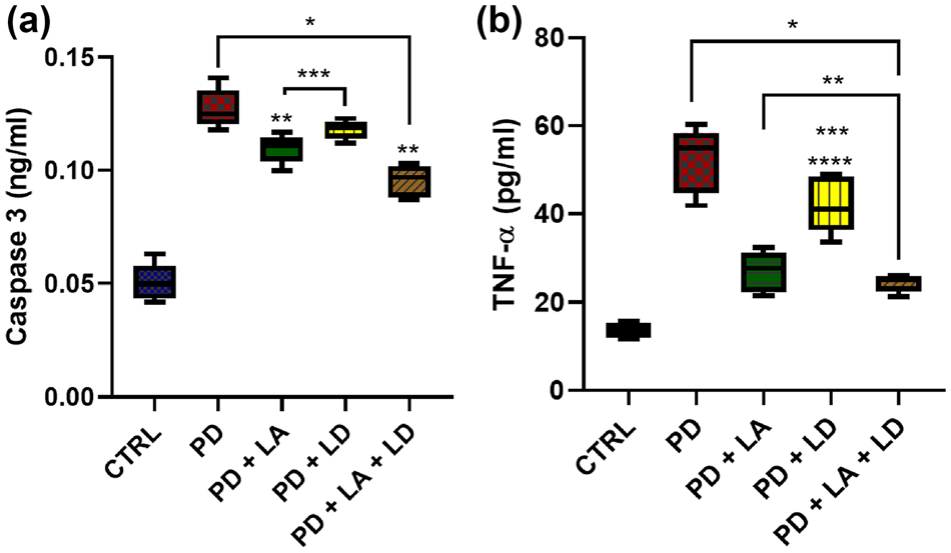
(a) Caspase‐3 expression and (b) tumor necrosis factor‐alpha (TNF‐α). Data are presented as mean ± SEM. *p < .05 compared to control; **p < .05 compared to untreated PD group; ***p < .05 compared to PD + LA + LD. LA, lauric acid; LD, levodopa; PD, Parkinson's disease.

All of the PD flies had significantly (*p* < .05) increased level caspase‐3 (Figure 5a) than control flies. Conversely, the flies in the PD + LA and PD + LA + LD groups displayed significantly (*p* < .05) reduced level of caspase‐3 compared to the untreated PD flies, whereas the PD + LD group did not. Additionally, there was no significant difference between the PD + LA and PD + LD groups, although the PD + LA and PD + LD groups had considerably (*p* < .05) higher caspase‐3 expression than the PD + LA + LD group (*F* (4, 16) = 98.22, *p* < .0001).

All PD flies showed considerably (*p* < .05) higher levels of TNF‐α than control flies, as seen in Figure 5b. However, compared to the untreated PD flies, the flies in all treatment groups showed significantly (*p* < .05) decreased TNF‐α level. The PD + LD group had a significantly higher level of TNF‐α compared to the PD + LA and PD + LA + LD groups, although there was no discernible difference between the PD + LA and PD + LA + LD groups (*F* (4, 16) = 45.31, *p* < .0001).

## 4 DISCUSSION

PD is a multifactorial and multicentric neurodegenerative disorder that is distinguished by a range of motor and non‐motor deficits (Pavelka et al., 2022). Accumulation and aggregation of α‐synuclein are the pathologic features that set PD apart from other forms of neurological disorders (Bridi et al., 2021). Synaptic dysfunctions and synaptopathy are associated with α‐synuclein aggregation prior to neuronal death (Calo et al., 2016). *Drosophila* was employed in the current study to determine the impact of LA and LD co‐treatment on α‐synuclein‐mediated Parkinsonism. To the best of our knowledge, this study is the first to suggest the ideal dose of LA to offer the most effective treatment of PD in the *Drosophila* model (250 mg/kg diet).

This study, to the best of our knowledge, is the first to report on how LA affects *Drosophila* models. The concentrations of LA that trigger therapeutic effects vary between 125 and 500 mg/kg diet. However, this study offers a unique suggestion that 250 mg/kg LA diet may be the dose of LA with optimal therapeutic impact on *Drosophila* PD model.

The findings of the present study showed that overexpression of α‐synuclein significantly decreased the locomotive capacity of the flies, which agrees with the results of Ikeda et al. (2019). The development of motor dysfunction may be caused by the disruption of the activity of α‐synuclein in neurotransmitter production (DeWitt & Rhoades, 2013), coupled with a buildup of aggregated α‐synuclein in the mitochondria of dopaminergic and serotonergic neurons, which may contribute to the neurons' susceptibility to oxidative stress and mortality (Ganjam et al., 2019; Haque et al., 2022). This outcome supports the finding that α‐synuclein aggregation is associated with motor neuron dysfunction and degeneration (Roberts et al., 2022). Olfactory dysfunction is one of the age‐related brain damage associated with PD (Avanipully, 2022). The findings from this study demonstrated olfactory deficits in the α‐synuclein *Drosophila* model of PD. Notably, this is the first study to report an olfactory deficit in the α‐syn/Ddc *Drosophila* model, to the best of our knowledge. One possible explanation for this is the misfolding of α‐synuclein and its subsequent accumulation within the cells of the olfactory bulb (Avanipully et al., 2022), thereby impairing neural trajectories needed for encoding and decoding olfactory sensation.

Impaired longevity in α‐synuclein transgenic *Drosophila* models is well established in literature (Ikeda et al., 2019; Yan et al., 2019). The shortened lifespan of the PD flies in this study is traceable to various factors including α‐synuclein‐mediated apoptosis, ferroptosis, and pyroptosis (Jung et al., 2023; Zheng et al., 2023). However, LA, LD, and their combination provided protection against the α‐synuclein‐induced locomotor and olfactory abnormalities and increased survival rates in the treated flies.

LA was able to improve motor and olfactory performance and prolonged longevity in the flies possibly due to its antioxidant properties (Anuar et al., 2023). Additionally, LA is known to possess the ability to increase the total ketone body concentration in the cell (Sheela et al., 2019). Ketone bodies give the neurons in the brain a non‐glucogenic form of energy that boosts oxidative mitochondrial metabolism (Veyrat‐Durebex et al., 2018), and protects and nourishes the neurons (McMullen et al., 2023), thereby promoting locomotor and olfactory activity, as well as extending the lifespan (Naithani & Karn, 2020; Bhoumik et al., 2023). LD improved motor functions by transforming to dopamine and increasing the amount of dopamine in the brain needed for the initiation and regulation of motor activities and survival (Ayikobua et al., 2020; van Vliet et al., 2023). It is also well elucidated in literature that the dopamine reward system promotes actions that extends lifespan by enabling concentration on important cues that will help meet survival needs including food, reproduction, and social interaction (Dreher et al., 2008; Kim et al., 2017; García‐Cabrerizo et al., 2021).

A major sign of cellular injury is the buildup of MDA contents, a secondary byproduct of lipid peroxidation (O. Idowu, Dosumu, et al., 2022; Shareef et al., 2022). PD‐related oxidative stress and apoptosis are triggered by neuronal overproduction of H_2_O_2_ (Deus et al., 2022; Xin et al., 2022). The increased levels of MDA and H_2_O_2_ found in this study support the hypothesis that overexpression of the α‐synuclein gene causes an excess of free radical production in genetic models of neurodegenerative diseases, which ultimately results in oxidative stress (Angelova, 2021). Previous reports have indicated the inhibition of antioxidant enzyme activity and a reduction in TAS in *Drosophila* models of PD (Siddiqueet al., 2021; Liu et al., 2022; O. K. Idowu et al., 2024). The overall downregulation of antioxidant capacity observed in the present study is traceable to the effects of α‐synuclein overexpression on the PD flies, which could be linked to mitochondrial dysfunction and excessive free radical production (Angelova, 2021). The quantity of healthy cells in a sample is used to estimate cell viability (Kamiloglu et al., 2020). According to the findings of this study, the decreased cell viability may be caused by α‐synuclein buildup in the neurons, leading to apoptosis (Huynh et al., 2023). Contrarily, LD and LA treatment resulted in a reduction in MDA and H_2_O_2_ levels, an improvement in SOD and GPx activity, a rise in the level of total antioxidants, and an increase in cell viability. This is attributable to the free radical scavenging property of LA (Srisuksai et al., 2024). The capacity of LA to boost the production of glutathione, and enhance mitochondrial metabolic activity and ATP generation could have been an added advantage (Nukaga et al., 2020; Alhamzah et al., 2023); hence, lowering oxidative damage (Zheng et al., 2023). Likewise, the antioxidant action of LD might be related to the production of dopamine (Stansley & Yamamoto, 2015), a powerful antioxidant that also shields neurocytes from oxidative stress by scavenging free radicals (Iuga et al., 2011).

Numerous cell types, including neurons, are found to undergo apoptosis due to elevated levels of misfolded α‐synuclein  (Sohrabi et al., 2023). Increased caspase‐3 activity has been linked to neuronal death in experimental models of neurodegenerative disorders (Dahmardeh et al., 2019; Rahul et al., 2020). In the present study, the PD flies exhibited elevated caspase‐3 activity. Tompkins et al. (1997) had previously attributed apoptotic activity to chromatin changes and deoxyribonucleic acid (DNA) fragmentation in the dopaminergic neurons, indicating that apoptosis plays a major role in the loss of neurons in PD. To maintain neuronal homeostasis, microglia and astroglia constantly monitor the brain parenchyma by secreting neurotrophic substances and pruning synapses. However, inflammation or toxic protein aggregates can over‐activate these glial cells, resulting in persistent neuroinflammation process that appears to be a co‐factor for disease progression (Provenzano et al., 2023). Overexpression of TNF‐α observed in this study may have resulted from the activation of astrocytes and microglia by aggregated neuronal α‐synuclein to produce the pro‐inflammatory cytokines (Yi et al., 2022). However, LA and LD therapy reduced caspase‐3 activity as well as TNF‐α expression. The anti‐apoptotic effect potentiated by LA is linked to the ability of ketone bodies to inhibit DNA breakdown and apoptotic signaling pathways in neurons (Chung et al., 2022). Although the exact mechanism by which LD inhibits caspase‐3 activation is not clear, it could have resulted from its inhibitory action on mitogen‐activated protein kinases (MAPK) (Sabens et al., 2011). Previously, it has been shown that dietary fatty acids can alter how TNF‐α is regulated and produced (Joffe et al., 2013). LA may have had an anti‐inflammatory impact by preventing nuclear factor‐kB activation and phosphorylating MAPK (Huang et al., 2014), although these are not assayed for in this study. The downregulation of the expression of TNF‐α by LD could have resulted from the inhibitory effect of dopamine on microglia and astrocyte activation (Zhao et al., 2014; Iliopoulou et al., 2021).

## 5 CONCLUSION

The results of this study show that LA and LD play an important ameliorative role in PD‐like features in *Drosophila melanogaster* model. This is evident in their modulation of neurobehavioral activities, reduction of oxidative stress, and regulation of apoptotic and inflammatory processes. The co‐administration of the two compounds also brought about a little better modulation of redox parameters, longevity, and apoptotic and inflammatory processes than either of the two compounds alone.

### 5.1 Limitation of the study

The outcomes of this study would have been strengthened by information on mitochondrial function and genetic changes, as well as microscopy data from particular brain regions, to bolster the molecular mechanism behind the neuroprotective effects of LA and LD.

## AUTHOR CONTRIBUTIONS

**Olumayowa K. Idowu**: Conceptualization; investigation; writing—original draft; resources; methodology; data curation. **Olufunke O. Dosumu**: Conceptualization; methodology; supervision; validation; writing—review and editing. **Ayodeji S. Boboye**: Investigation; formal analysis; writing—review and editing; visualization; data curation. **Ademola A. Oremosu**: Investigation; validation; methodology; supervision; writing—review and editing. **Abdullahi A. Mohammed**: Writing—review and editing; visualization; formal analysis; investigation; software.

### PEER REVIEW

The peer review history for this article is available at https://publons.com/publon/10.1002/brb3.70001

## Data Availability

The data that support the findings of this study are available from the corresponding author upon reasonable request.
